# Groundwater nitrate response to sustainable nitrogen management

**DOI:** 10.1038/s41598-017-07147-2

**Published:** 2017-08-17

**Authors:** Birgitte Hansen, Lærke Thorling, Jörg Schullehner, Mette Termansen, Tommy Dalgaard

**Affiliations:** 1 Geological Survey of Denmark and Greenland, Department of Groundwater and Quaternary Geology Mapping, 8000 Aarhus C, Denmark; 2 Aarhus University, Department of Environment Science – Environmental Social Science, 4000 Roskilde, Denmark; 3 Aarhus University, Department of Agroecology – Agricultural Systems and Sustainability, 8830 Tjele, Denmark

## Abstract

Throughout the world, nitrogen (N) losses from intensive agricultural production may end up as undesirably high concentrations of nitrate in groundwater with a long-term impact on groundwater quality. This has human and environmental health consequences, due to the use of groundwater as a drinking water resource, and causes eutrophication of groundwater-dependent ecosystems such as wetlands, rivers and near-coastal areas. At national scale, the measured nitrate concentrations and trends in Danish oxic groundwater in the last 70 years correlate well with the annual agricultural N surpluses. We also show that the N use efficiency of agriculture is related to the groundwater nitrate concentrations. We demonstrate an inverted U-shape of annual nitrate concentrations as a function of economic growth from 1948 to 2014. Our analyses evidence a clear trend of a reversal at the beginning of the 1980s towards a more sustainable agricultural N management. This appears to be primarily driven by societal demand for groundwater protection linked to economic prosperity and an increased environmental awareness. However, the environmental and human health thresholds are still exceeded in many locations. Groundwater protection is of fundamental global importance, and this calls for further development of environmentally and economically sustainable N management in agriculture worldwide.

## Introduction

Today more than half of the world’s population is nourished by agricultural outputs that have been produced using artificial fertilizers^1–3^. This became possible in the early part of the 20th century thanks to the invention of the Haber-Bosch process, which converts inert atmospheric free nitrogen (N) into reactive ammonia. An excess of reactive forms of N from agricultural production has caused an anthropogenic disturbance of the global N cycle, which threatens the stability of the planet^4–7^ due to an unintended cascade of N emissions and reactions in the air and the aquatic environment, ultimately also affecting human health^1^. This includes leaching of nitrate to groundwater and the appearance of nitrate as a common pollutant in the upper oxic and anoxic nitrate-reducing parts of aquifers^8^.

Protection of groundwater is of fundamental global importance due to its many uses, for example as drinking water and in industrial and agricultural productions, and because of its environmental value for groundwater-dependent ecosystems such as wetlands, rivers and near-coastal areas^9^. The groundwater and drinking water standards are set to a maximum of 50 mg l^−1^ nitrate in the European Union (EU) following the recommendations of WHO^10^, which is almost equal to the US Environmental Protection Agency’s maximum contaminant level of 10 mg l^−1^ N^11^. Nitrate thresholds values in groundwater in regard to surface water pollution might be well below the drinking water nitrate standard dependent on the specific sensitivity of the ecosystems^12^. The drinking water standard is based on clinical epidemiological evidence and aims to protect infants from the acute condition called methemoglobinemia^10^. However, the endogenous nitrosation of nitrate into nitrite and reaction of nitrite with nitrosatable compounds into carcinogenic N-nitroso compounds are suspected to lead to adverse chronic effects such as colorectal cancer^13^ and bladder cancer^14^. In the EU, about 75% of residents depend on groundwater for their water supply^15^. In many countries throughout the world the supply of fresh water has become inadequate due to a growing population and increasing industrial and agricultural production in a changing climate^16, 17^.

Denmark has implemented EU directives (Nitrates Directive, 1991/696/EC; Water Framework Directive, 2000/60/EC and Groundwater Directive, 2006/118/EF) into the national legislation, and has been introducing several policy action plans since 1985 to protect groundwater and surface waters from unsustainable N losses from mainly agriculture.

Agricultural development in Denmark has historically contributed substantially to economic growth and societal prosperity. Especially during the past decades, this has resulted in a considerable growth in plant and animal inputs and outputs, improved farming N use efficiency, and structural changes towards larger and more intensive farms, especially in the livestock sector.

Intensive agricultural production is a threat to groundwater quality due to N leaching from soils to groundwater. Therefore, nitrate present in groundwater in rural areas is strongly linked to the amount of N applied to agricultural land, and to the N surplus in particular^18^. The resulting nitrate concentration in groundwater depends on 1) net precipitation and nitrate leaching from land use, 2) redox conditions and nitrate reduction for example with pyrite oxidation in the groundwater system^19, 20^, and 3) hydrogeological conditions and residence time in the groundwater system^21^. Long-term effects of N losses from agricultural land on groundwater nitrate content have been reported from all over the globe^22–26^. In some parts of the Western world improved N management has significantly decreased the nitrate content in groundwater, although it often still does not meet groundwater and drinking water standards in all areas.

The knowledge and technology exist to further lower the N impact from agriculture on the aquatic environment including groundwater quality by improving the efficiency of N use both in the Western world and globally. This can be accomplished by increasing the utilization of nutrients applied to agricultural land, but often social and economic factors have hindered the adoption of effective measures in policy implementation^27^. However, there is also evidence to suggest that socio-economic development actually stimulates adoption of environmental protection measures and that economic growth can curb environmental degradation. In the context of N emissions, different measures have been suggested. These include the use of the 4 R principle (right time, right source, right timing, and right placement)^27^ and the use of N-footprints to encourage consumers to practice N sustainable behaviour, for example by reducing food waste, increasing recycling and consuming less meat^28–30^.

We use measurements of nitrate in Danish groundwater representing the past 70 years to analyse the N sustainability of intensive agricultural N management in relation to groundwater protection and economic growth. This type of analysis has previously been applied to other environmental quality indicators^2, 31^, but to our knowledge this has not been done for long-term groundwater nitrate measurements.

## Groundwater protection and N regulation in agriculture

**Figure 1:**
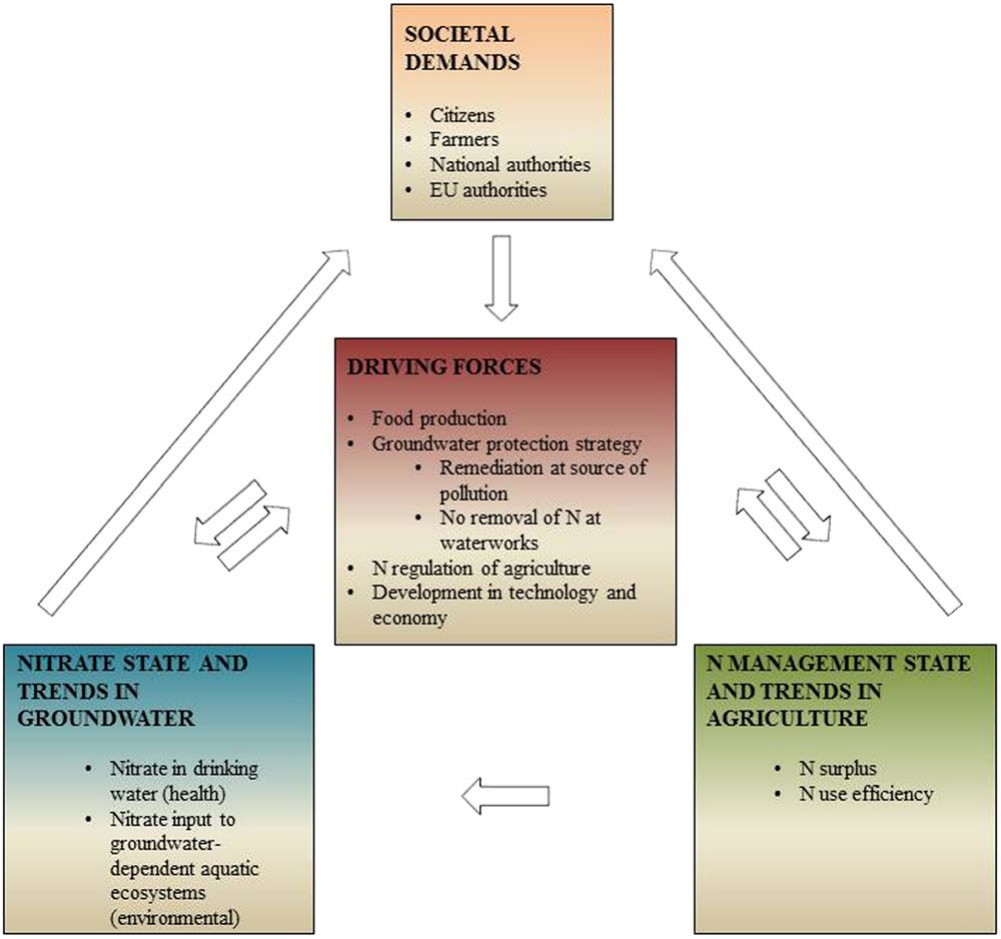
Schematic of the relationship between nitrate response in ground water, sustainable N management in agriculture and major societal demands and driving forces, as inspired by the case of Denmark.

In agricultural areas, nitrate in groundwater is a direct response to farm and field N management due to the leaching of nitrate from the root zone. Societal demands for groundwater protection have pushed the use of N in agriculture in a more environmentally sustainable direction. This means that N management in agriculture is linked to nitrate state and trends in groundwater. This interdependency is schematically shown in Fig. 1, as inspired by the case of Denmark.

National and European policy initiatives (Nitrates Directive, Water Framework Directive, and Groundwater Directive) on groundwater protection (and often surface waters as well) from agricultural N losses are important drivers for sustainable N management in agriculture. Since the mid-1980s, Danish agricultural N regulation has mainly been addressed at a national level, and a large array of measures have been imposed to protect groundwater; for example: maximum livestock density, prescriptions for the handling of manure, compulsory growing of catch crops on a percentage of agricultural land, a maximum N allowance for crops that is below the economically optimal application, subsidies for afforestation, conversion to organic farming, etc (Table 1).^32^.

**Table 1:** The major Danish N policy measures implemented over the past 31 years with the Danish action plans (AP) in 1985 (NPo), 1987 (AP-1), 1991 (AP for a more sustainable agriculture), 1998 (AP-II), 2001 (ammonia AP), 2004 (AP-III), 2009 (Green Growth AP) and 2016 (Food and agriculture package AP) (updated from Dalgaard *et al*.^32^).

Year	N measures imposed	N measure abandoned
**1985**	Max. stock density. Mandatory slurry tank floating barriers. No runoff from silage clamps and manure heaps. Min. slurry capacity and ban on winter spreading of slurry for spring crops.	
**1987**	Mandatory fertilizer and crop rotation plans. Min. proportion of area with winter crops. Mandatory manure application within 12 h.	
**1991**	Statutory norms for fertilizer N application for specific crops. Max. N applied to crops equalling economic optimum. Subsidies to low-N grasslands in environmentally sensitive areas.	
**1998**	Max. N applied 10% below economic optimum. 6% obligatory catch crops. Subsidies to more organic farming, wetlands, extensification and afforestation. Sites-specific groundwater protection zones	
**2001**	Promotion of low excretion livestock feeding.	
**2004**	More catch crops. Tightened ammonia restriction (e.g. broadcasting banned), and special restrictions near sensitive nature areas. Subsidies to promote better manure handling and animal housing.	
**2009**	10 m buffer zones around streams, lakes and sensitive habitats. Max. N applied ≈ 15% below the economically optimal production. Optimized feed practice promotion.	
**2016**	Less national N regulation, but more spatial differentiated N regulation with locally targeted measures as e.g. constructed wetlands and additional catch crops.	10 m buffer zones (back to 2 m). Max. N applied ≈ 15% below the production economic optimum (back to production at economic optimum).

In Denmark, protection of groundwater is given a high priority in order to secure sufficient drinking water of high quality and to obtain a satisfactory low impact on groundwater-dependent aquatic ecosystems. This has been driven by a long-term, stable, national groundwater protection strategy demanding remediation of groundwater pollution threats at the source of the pollution (mainly agriculture) to ensure that only simple treatment at the waterworks, such as aeration and filtration, will be necessary, since removal of N at the waterworks is not a politically acceptable option. At the same time, intensive agricultural plant and livestock production systems are pushed towards more sustainable N management with lower N surpluses and higher N use efficiency (NUE) while increasing production. This sustainable N management is influenced both by agricultural N regulations and by general developments in technology and the economy.

## Local variation in nitrate response

The effect of national N regulation and agricultural N losses on groundwater quality is evaluated here by analyzing long-term time series of nitrate measurements in oxic groundwater at individual monitoring points. We focused on oxic groundwater due to the stability of nitrate in the presence of oxygen. At the monitoring points, groundwater age is also measured because it has proven to be an essential component of groundwater trend investigations. Its inclusion allows concentrations of nitrate to be related to the time of recharge instead of the time of sampling. In this way, comparison becomes possible between nitrate in groundwater and N leaching from agriculture^33^.

**Figure 2:**
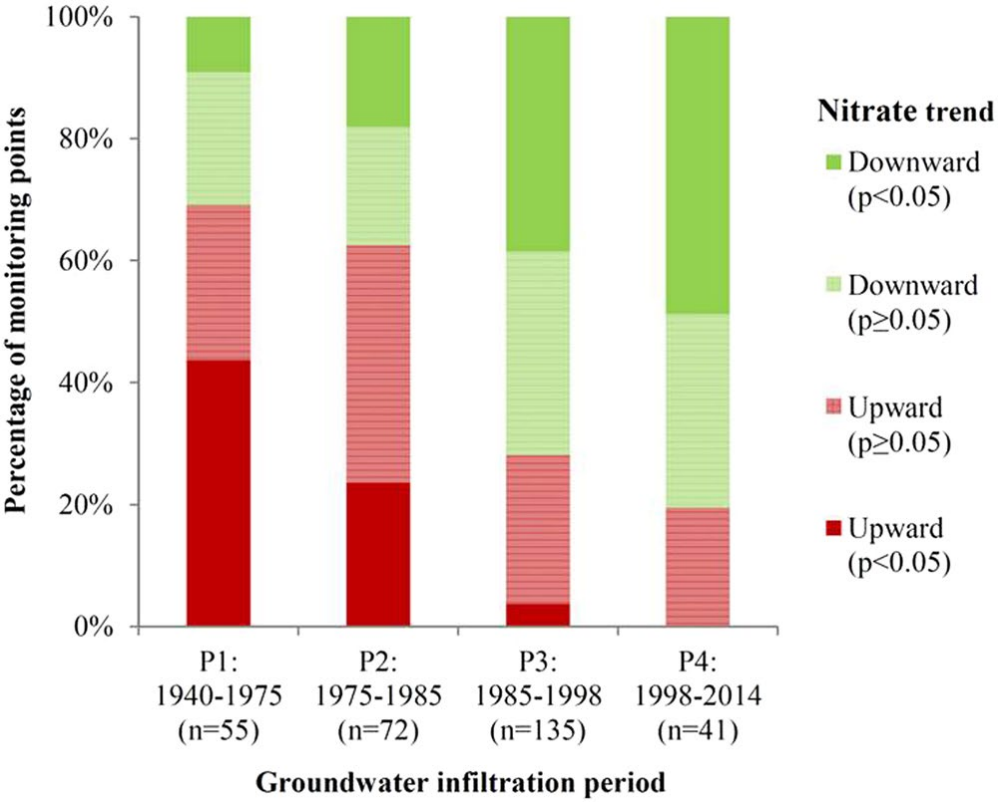
Development in nitrate trends in oxic groundwater based on linear regression of nitrate time series in 250 individual monitoring wells. Trends are divided into four groups according to direction of the trend (slope of the regression line) and four groundwater infiltration periods (P1 – P4). Both significant (p < 0.05) and non-significant (p ≥ 0.5) trends are shown. The numbers of monitoring wells in each period are shown (n).

Time series of nitrate concentrations in oxic groundwater were examined with linear regression analysis, taking groundwater infiltration time into account. The analysis included a total of 3,233 nitrate measurements from 250 monitoring points from rural areas all over Denmark, each providing data for a minimum of eight years. A time span of more than 70 years of groundwater nitrate response shows the cumulative result of 303 calculated nitrate trends divided into four groundwater infiltration periods: P1: 1940–1975, P2: 1975–1985, P3: 1985–1998 and P4: 1998–2014 (Fig. 2). There is the highest representation of data in the two middle periods (P2 and P3), due to an unequal distribution of groundwater ages at the selected monitoring points. The respective lengths of the four periods were based on the overall development of the nitrate content in oxic groundwater and the timing of various Danish environmental action plans and major N policy measures presented in Table 1 ^32^.

A clear overall tendency can be seen for a shift from broadly increasing to broadly declining nitrate trends over time, both when the development of the significant trends is considered and when significant and non-significant trends are examined. In the first groundwater infiltration period (P1: 1940–1975), almost 70% (25% is non-significant, p ≥ 0.05) of the monitoring points show upward nitrate trends, in contrast to the last period (P4: 1998–2014) where it applies to only around 20% (all non-significant, p ≥ 0.05). Consequently, over time an increasing number of the monitoring points show downward nitrate trends, indicating that N regulation of Danish agriculture has had a clear effect on reducing nitrate concentrations in oxic groundwater.

**Figure 3:**
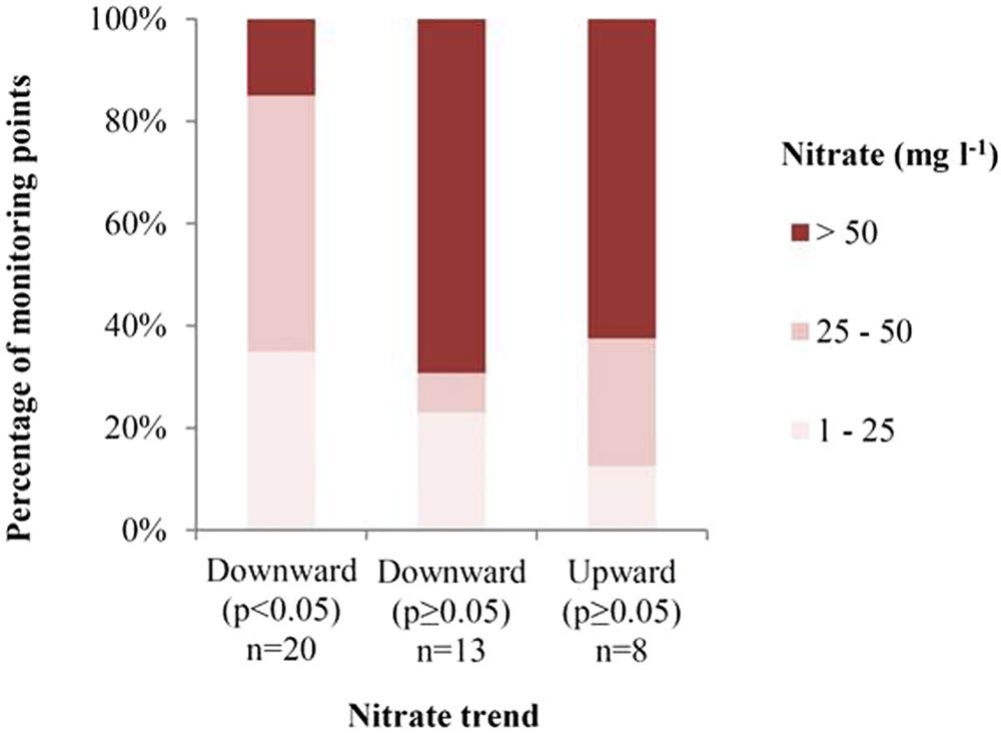
Latest measured nitrate concentration (2014) illustrated in three oxic groundwater nitrate classes (>50 mg l^−1^, 25–50 mg l^−1^ and 1–25 mg l^−1^) grouped according to the nitrate trends in the groundwater infiltration period P4: 1998–2014.

In order to evaluate the current state of the groundwater, the nitrate concentrations and trends in the latest period (P4: 1998–2014) were considered. The current status shows that nitrate concentrations have been falling, but that approx. 40% of the monitoring points in oxic groundwater still have concentrations above the drinking water standard of 50 mg l^−1^ nitrate (Fig. 3). The monitoring points with significant downward trends had the lowest nitrate concentrations at approx. 15% above the 50 mg l^−1^ nitrate limit, indicating that N regulation is making a difference at these locations. However, the highest concentrations were observed in the two groups of non-significant downward and upward trends. This indicates that further improvement of current N management and regulation is needed to reduce the nitrate concentrations in a clear downward direction at all locations to comply with current standards.

## National groundwater nitrate concentrations and agricultural influence

**Figure 4:**
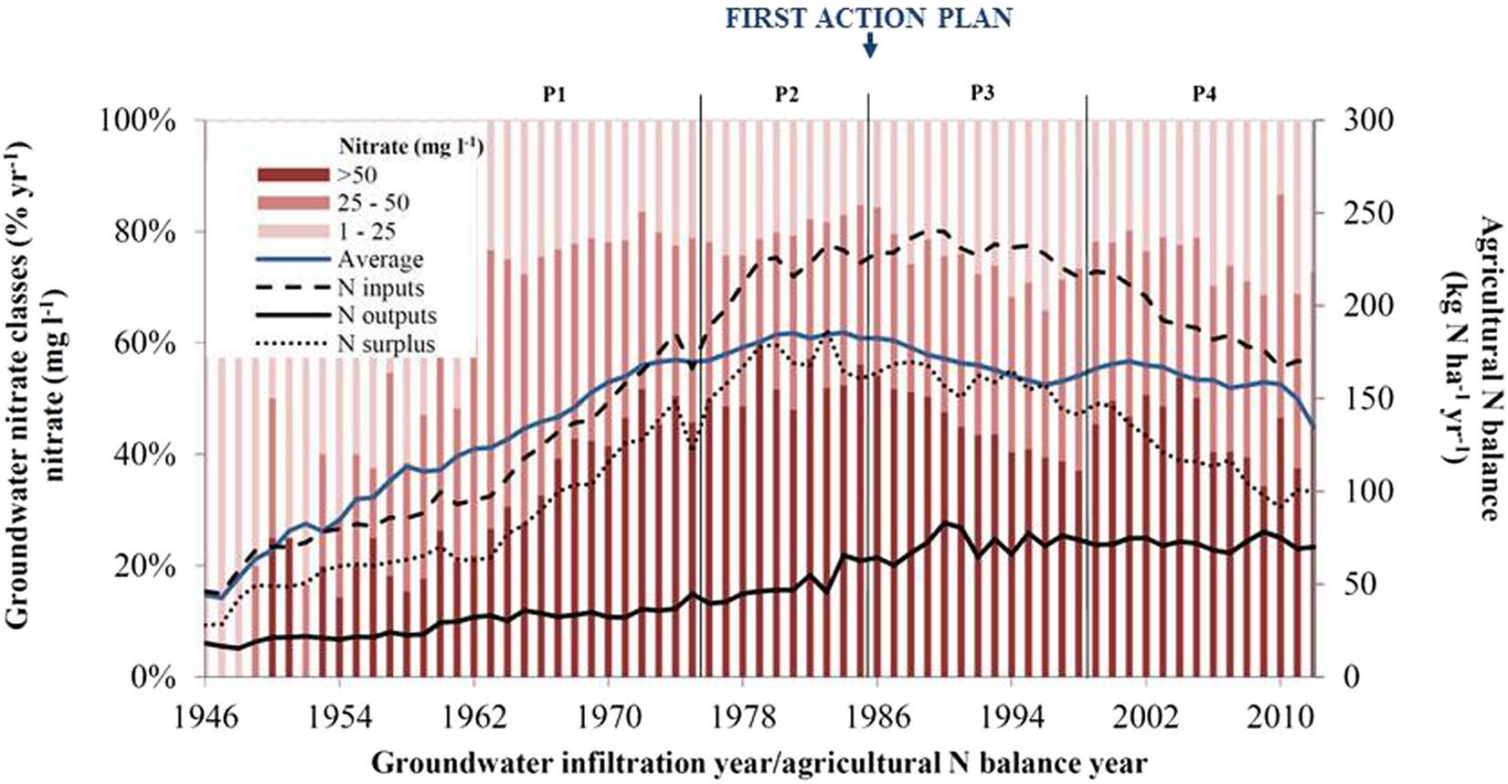
The annual trends of nitrate in oxic groundwater and agricultural N balances for Denmark in the four groundwater infiltration periods (P1-P4). The percentage of samples for each year is illustrated in the three groundwater nitrate classes (>50 mg l^−1^, 25–50 mg l^−1^ and 1–25 mg l^−1^). The 5-year moving average of the nitrate content in oxic groundwater (blue line) is based on 5,506 samples from 340 monitoring points. The annual agricultural N balances, net (N inputs, outputs and surplus) are calculated for the primary Danish agricultural sector.

**Figure 5:**
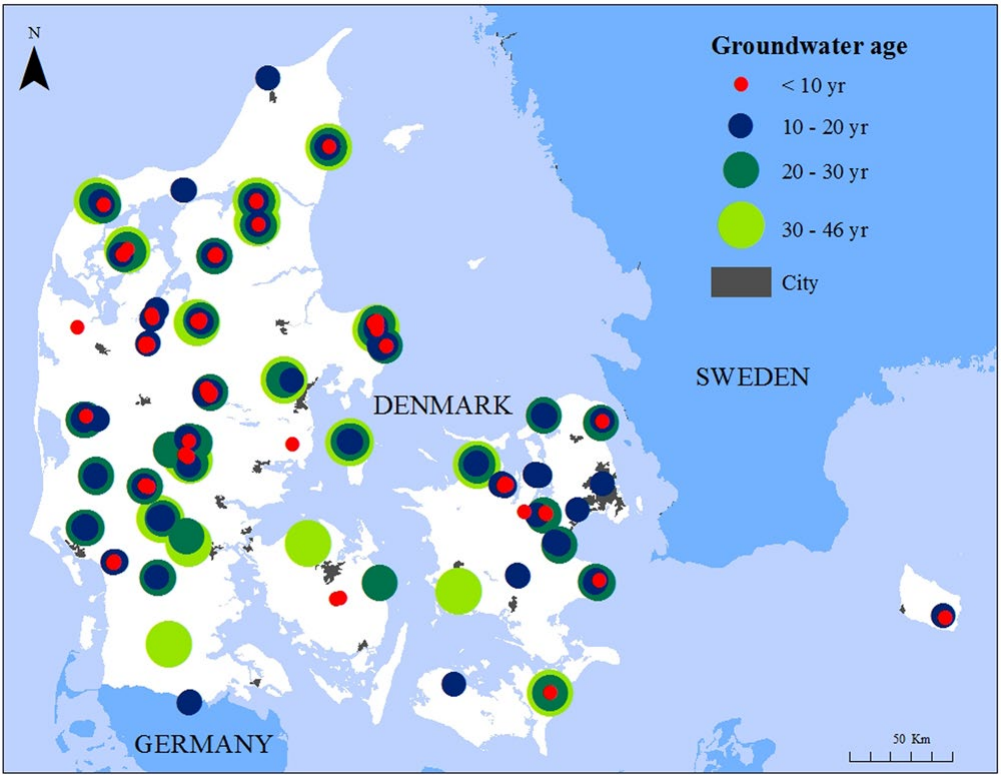
The geographic location of the 340 monitoring points in oxic groundwater in rural areas of Denmark, and the age of the studied groundwater. The map was created by ArcGIS 10.2.2 (www.esri.com).

Evaluation of the effect of national N regulation on groundwater quality is also performed by compiling all long-term groundwater monitoring nitrate time series into one single national analysis, taking the groundwater infiltration time into account. In Fig. 4 the distribution and development in nitrate concentrations in oxic groundwater, based on 5,506 samples from 340 monitoring points in agricultural areas all over Denmark (see Fig. 5), are compared with the development in agricultural N balances. These are based on the national N inputs (mainly synthetic fertilizer and import of animal feed), and N outputs from the sector (production of plant and animal products) in Danish agriculture from 1946–2012.

**Figure 6:**
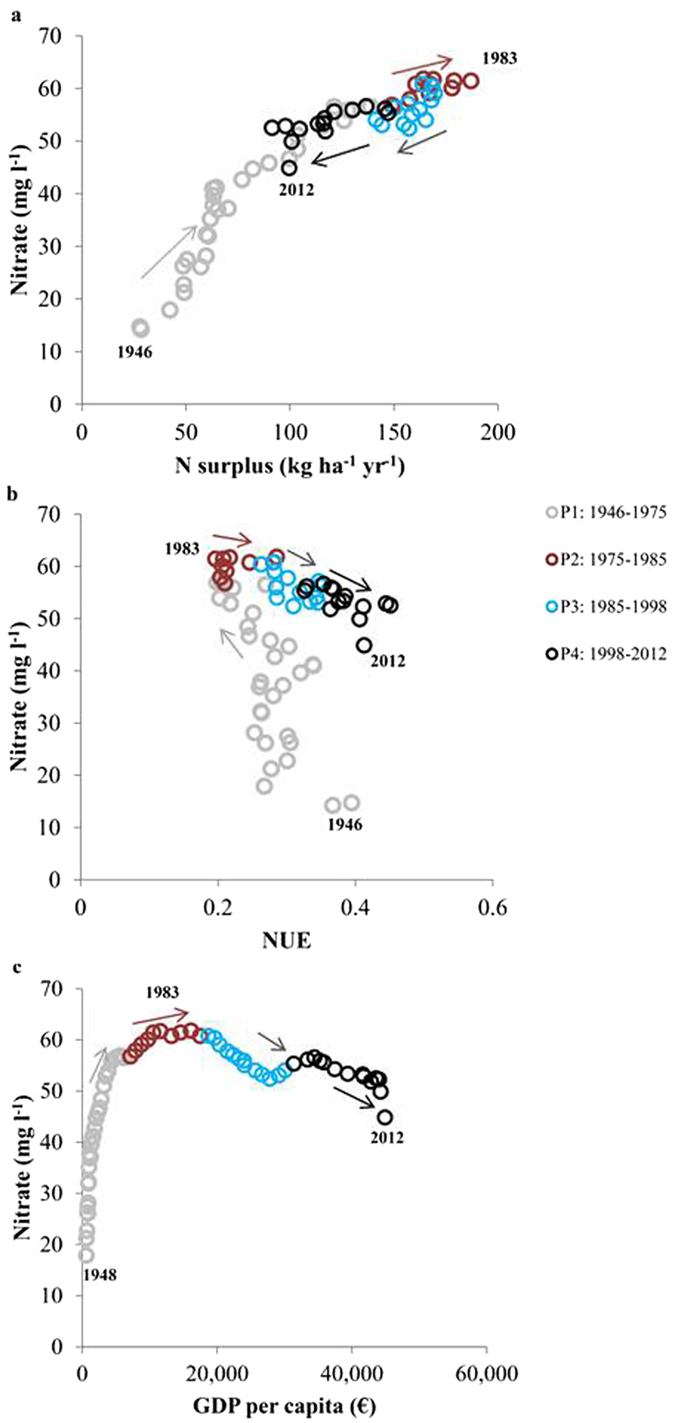
Nitrate trend reversals in oxic groundwater in the four groundwater infiltration periods. a, Annual N surplus in Danish agriculture. b, Annual N use efficiency (NUE) in Danish agriculture. c, Annual Danish gross domestic product per capita (GDP per capita, current prices) in euro (€).

N surplus is calculated annually as the difference between agricultural N inputs and outputs, and is the amount of N not directly used in the agricultural production and therefore at risk of being lost to the environment, for example in the form of nitrate leaching to groundwater. There is a clear correlation between the annual development in N surplus and the national concentration of nitrate in oxic groundwater (Fig. 4). Like the N surplus, the nitrate content reached a maximum around the previously detected turning point at the beginning of the 1980s^8, 33^, as also seen in other countries in the Western world^18, 27, 34^. Since the trend reversal, both the nitrate content in oxic groundwater and the N surplus have been declining. However, the amount of nitrate analyses per year in the highest class (>50 mg/l) has increased in the latest period (P4: 1998–2014). This might be due to a higher uncertainty because of lower data coverage in P4 compared to the former two periods (see Figs 2, 4 and 6).

Danish agriculture has a high fertilization rate and livestock density compared with European and global averages^35^. N inputs to Danish agriculture increased from 1946 until 1990 from about 46 to 240 kg N ha^−1^ yr^−1^. By 2012, the N inputs had declined to around 170 kg N ha^−1^ yr^−1^. Different factors might have influenced the decrease in N input, as changes in land use. However, it is most likely that it is due to a drop in the use of synthetic fertilizer due to a better N use efficiency stimulated by the different N mitigation measures in the environmental action plans (Table 1). These N mitigation measures include better management and utilization of N in animal manure, statutory norms for fertilizer N application for specific crops, maximum limits on N applied to specific crops under economical optimum levels, and increasing use of catch crops.

In line with the increasing N inputs, the N outputs also increased from 1946 to 1990, from about 18 to almost 80 kg N ha^−1^ yr^−1^ due to increased agricultural production. The increased agricultural production since the 1940s, has been stimulated by the growing N inputs of synthetic fertilizers and imported feed for the animal production. Especially the production of pigs has increased dramatically from 3.2 million animals per year in 1950 to 31.7 million in 2016^36^.

Since 1990, the output of N has been nearly constant in Danish agriculture, even though N inputs and N surplus have been declining. This is mainly due to a better utilization of N in animal manure and a shift towards a higher livestock production.

## Groundwater nitrate response to growth and sustainability

On an annual national basis, the effect of agricultural N management on nitrate concentrations in oxic groundwater was also evaluated by analysing the relationship between nitrate in oxic groundwater (5-year moving average) and annual N surplus, N-use efficiency (NUE) and the gross domestic product, GDP (Fig. 6). Here, the analyses included the same data as shown in Fig. 4, where the data is divided into the four previously mentioned groundwater infiltration periods (Fig. 2). Linear regression analyses showed that the three correlations for the four groundwater infiltration periods in Fig. 6 were all statistically significant (p < 0.05). The arrows in Fig. 6 illustrate whether there is an increase or decrease with time.

Overall the analyses in Fig. 6(a,b) support a trend reversal of nitrate in oxic groundwater around 1983 related both to the reduction in agricultural N surplus and the increase in N use efficiency. The relationship between nitrate in oxic groundwater and GDP supports an inverted U-shape with a turning point in 1983 (Fig. 6(c)). This is consistent with the EKC (Environmental Kuznets Curve) hypothesis that environmental degradation is curbed by economic growth^37^.

In the first period (P1: 1946–1975), the N surplus increases every year from 28 (1946) to 121 kg N ha^−1^yr^−1^ (1975). Groundwater responds with an increase in nitrate from 15 to 56 mg l^−1^ for the period. In the same period, the agricultural use of N became increasingly environmentally unsustainable. The NUE decreases from 40% to about 20%. Denmark experienced economic growth, the GDP per capita increased from approx. 600 to 6,200 € from 1948 to 1975 (data is not available for the first part of the period). A growing agricultural production was sustained by a heavier reliance on synthetic fertilizers.

In the second period (P2: 1975–1985), environmental issues became more prominent in the political debate, the Danish Ministry of Environment was formed in the early 1970s and the first national environmental action plan was initiated in 1985. Significant turning points are seen around year 1983 with regard to both N surplus, NUE and the responding concentrations of nitrate in groundwater. The N surplus levels out and fluctuates around 150 to 185 kg N ha^−1^ yr^−1^. Nitrate concentrations in oxic groundwater are at a maximum of around 57–62 mg l^**−1**^. The NUE starts to increase from 20 to 28%, while the economic growth continues to increase (GDP per capita increased from approx. 7,200 to 17,500 €).

In the third period (P3: 1985–1998), environmental action plans were implemented, and a more sustainable N management was initiated and reflected in the decreasing N surplus (from 170 to 141 N ha^−1^ yr^−1^), and further increases in N utilization reflected in increases in NUE from 28 to 34% as yearly averages. The more sustainable N management results in significant reductions in nitrate concentrations to oxic groundwater from around 61 to 54 mg l^**−1**^ as 5-year moving average values consistent with the EKC (Environmental Kuznets Curve) hypothesis^37^.

In the fourth and most recent period (P4: 1998–2012), environmental protection continues and new environmental action plans and measures are implemented, while economic growth also continues. GDP per capita increases from approx. 31,400 to 44,900 €, and NUE increases from 33 to 41%. However, the development shows a lower rate of reduction in nitrate concentrations (from 55 to 45 mg l^**−1**^) in oxic groundwater, while the N surplus continues to decrease (from 147 to 100 N ha^−1^yr^−1^) compared to the previous period.

## New sustainable paradigm for N regulation?

Our analysis shows that almost 30 years of N regulation in Danish agriculture have resulted in a clear reduction of nitrate concentrations in oxic groundwater. This trend has mainly been driven by societal demands for groundwater as well as surface water protection. Currently, the Danish N regulation is undergoing changes from a mainly one-size-fits-all national regulation with restrictions on the overall N application level towards a system that is to a larger extent based on locally targeted measures. Groundwater protection requires use of effective mitigation measures to minimize the N leaching from agricultural fields, e.g. extensive use of catch crops, or restrictions on the N application level to specific crops. However, in order to obtain a sufficient groundwater protection level with the new Danish N regulation there is a need for development of new hydro-geochemical mapping tools^21, 38^ in order to be able to identify the most N sensitive agricultural fields at a very fine spatial scale (hectares-scales). Introduction of locally targeted N measures is also challenged by other factors such as: prices of land, farmers’ resistance, challenges related to reallocation of land use between farms, collaboration of farmers, citizens and authorities, as well as the uncertainty on the local hydro-geochemical vulnerability assessment of the fields.

Other studies in the Western world also argued that stricter national regulations and regionally differentiated mitigation options are needed to further reduce the N impact on groundwater resources, but implementation of new mitigation options are impeded by social, economic and political factors^27, 34, 39^. Various new mitigation options have been proposed such as: 1) redistribution of N abatement obligations between farmers, 2) targeting efforts to local conditions, 3) improving NUE, 4) increasing recycling, 5) reducing food waste, 6) changing personal dietary choices, 7) partnerships between stakeholders, 8) education, 9) using performance-based indicators at farm level, 10) investments and 11) closing yield gaps on underperforming lands by global redistribution of N^4, 7, 27, 30, 34, 39–41^.

The current Danish paradigm shift in N regulation is mainly motivated by the necessity for continued increase in agricultural productivity^42^. However, the trends in the data suggest that the agricultural sector has been able to adjust to societal demands and to innovate agricultural production methods. Given the clear evidence of the impact of past environmental regulation, it seems safe to hypothesise that a relaxation of the regulation of agricultural N applications would result in an increase in nitrate concentrations in oxic groundwater. This will push nitrate in groundwater resources further towards the risk level for environmental and human health and increase the number of locations where the thresholds are exceeded delaying compliance with groundwater legislative requirements.

The presented 70 years Danish national analysis clearly demonstrates that agricultural sustainable N management affects nitrate concentrations in oxic groundwater. Our study highlights the importance of a consistence national groundwater monitoring programme, and the need for development of future effective N mitigation measures in intensive agriculture worldwide in order to protect groundwater resources.

## Methods

### Danish climate, geology, and agricultural land

Denmark is a small northern European country in Scandinavia with a total area of 43,000 km^2^, and a population of 5.7 million in January 2017. The glacial landscape of Denmark has a modest topography with the highest point 170 m above sea level, and a coastal temperate climate with precipitation between 600 and 1000 mm yr^−1^. The upper groundwater containing geological layers consist of 50–200 m thick quaternary deposits which are underlain by tertiary unconsolidated deposits or Cretaceous limestone and chalk. The agricultural use of the total Danish land has been steady decreasing from 75 to 63% during the last 70 years.

### Environmental action plans and N policy measures

The first Danish environmental action plan was launched in 1985 followed by several others in the following years (Table 1) with the overall aim of reducing N losses from agriculture to air, soil and water. The N policy measures in the action plans were implemented as national regulation from the beginning with equal norms and standards for all parts of the country followed by more sites specific measures related to e.g. environmental sensitive areas, organic farming, wetlands, afforestation or groundwater protection zones. In 2016 a new paradigm for Danish N regulation of agriculture was initiated with allowance of a higher N application rate, and more local target measures as e.g. constructed wetlands and additional use of catch crops on the fields in autumn and winter.

### Groundwater nitrate concentrations

Nitrate concentrations in groundwater were retrieved from the Danish Groundwater Monitoring Programme^43^, which has been running since 1988 in order to evaluate the effect of both national and European legislation on groundwater protection. Normally, the wells are sampled annually for nitrate as well as for other major chemical components (chloride, sulphate, ammonium, iron, etc.), pesticides and their metabolites, while trace elements (arsenic, copper, nickel, etc.) and organic pollutants are sampled at a lower frequency.

During groundwater sampling, online field measurements of pH, redox potential, oxygen concentration, temperature and conductivity were performed in order to ensure a high analytical quality and to produce representative groundwater samples according to Danish technical standards. The groundwater samples were analysed by professional, certified laboratories^44^.

The groundwater chemical concentrations are publicly available in the Danish national geodatabase on the website: www.geus.dk.

### Groundwater dating

Previous studies^33, 45^ have demonstrated that the age of groundwater (residence time) should be included as a component of groundwater trend investigations. Its inclusion may help to link changes in land use and N management practices to changes in groundwater nitrate concentrations. Groundwater age determination allows concentrations of nitrate to be related to the time of recharge instead of the time of sampling, which, in turn, makes comparison between nitrate in groundwater and N loss from agriculture possible.

CFC (chlorofluorocarbon) gases in the atmosphere arise from pollution with Freon gases, and atmospheric concentrations of CFCs rapidly increased between 1940 and the 1990s. However, as a result of the Montreal Protocol on Substances that Deplete the Ozone Layer adopted in 1987, atmospheric concentrations are now declining, and Danish results show that it is only possible to use the CFC method for dating groundwater recharge before year 2000^46^. Therefore, the tritium-helium-method was introduced in 2013 as a new method to date younger oxic groundwater in the Danish Groundwater Monitoring Programme.

Determination of groundwater recharge ages with the CFC-method followed the procedure of Busenberg & Plummer^47^, and Laier^48^. The analyses were performed at the laboratory of the Geological Survey of Denmark and Greenland (GEUS). The tritium-helium-method used to determine the groundwater recharge time after year 2000 followed the procedure of Gardner & Salomon^49^ and Sültenfuss^50^, and the analyses were performed at the Institute of Environmental Physics, University of Bremen.

In this study the CFC-method and the tritium-helium-method were used to date groundwater recharge from 1940–2000, and from 2000–2014, respectively. Under optimal conditions both methods allow determination of groundwater ages with an analytical uncertainty of ±2 years^47–50^. A deep unsaturated zone and local areas with anoxic redox conditions might increase the uncertainty of the groundwater recharge determinations. However, here the focus is on oxic groundwater where the methods are most reliable due to stability under oxic conditions. In addition, studies on sandy soils in Denmark have shown that the resistance time of water in the unsaturated zone using tritium dating is equal to that of air diffusion in the unsaturated zone using the CFC-method^48^.

Groundwater recharge age was determined at least once at each groundwater monitoring point. Sampling years were converted to groundwater recharge years assuming a constant groundwater age at each groundwater sampling point. The recharge year was calculated for each yearly nitrate measurement as:

$${\rm{Groundwater}}\,{\rm{recharge}}\,{\rm{year}}={\rm{Sampling}}\,\mathrm{year}\,\div{\rm{Groundwater}}\,{\rm{age}}$$

The groundwater dating data is, like the nitrate analyses, publicly accessible in the Danish geodatabase on the website: www.geus.dk.

### Pre-processing of groundwater data

This study only includes groundwater monitoring points with 1) oxic groundwater, 2) age-dated groundwater and 3) more than eight years of nitrate measurements.

Only groundwater monitoring points in oxic groundwater were included because nitrate is degraded in anoxic groundwater. In addition, Danish oxic groundwater always contains nitrate due to impact from intensive farming all over Denmark. Due to its stability, nitrate in oxic groundwater is used as an indicator of nitrate leaching from the root zone and thus of losses of N from agricultural practices. Systematically, the water samples from oxic groundwater have been selected based on specific concentrations of the redox-sensitive parameters: nitrate (>1 mg l^−1^), iron (<0.2 mg l^−1^), and oxygen (>1 mg l^−1^)^33^. These criteria minimize the uncertainty in the determination of the groundwater redox state. Iron in groundwater is thus used as an indicator of complete nitrate reduction. The concentration levels of the other redox parameters (nitrite and manganese) show that an oxygen concentration higher than 1 mg l^−1^ is sufficient to ensure oxic conditions. This is possible due from to the high quality of the oxygen field measurements of the groundwater^8, 33^.

Only monitoring points with dated groundwater were included because these measurements enable nitrate sampling years to be converted to groundwater recharge years. In this way, it becomes possible to compare long-term changes in N surplus in agriculture with changes in oxic groundwater nitrate concentrations.

The third criterion addresses the length of the time series, as a minimum of eight years of nitrate analyses per monitoring point are needed in order to be able to perform a time series analysis, as recommended in Guidance Document No. 18^51^.

In the study, 340 groundwater monitoring points with oxic dated groundwater and with annual nitrate analyses from more than eight years per monitoring point were included in the national analysis (Fig. 5). The analysis was based on a total of 5,506 nitrate analyses sampled from 1988–2014, and representing groundwater recharged in the period from 1944–2014 presented in Figs 4 and 5. In the nitrate time series analysis performed in individual monitoring points (Figs 2 and 3), 250 monitoring points were included counting 3,233 nitrate analyses.

The Danish groundwater monitoring program has continuously been adapted to scientific, environmental, economic, legislative and political demands during the last 25 years. This means that the data from the program is not entirely uniform although consistency in time series from each monitoring wells has had a high priority. The 340 monitoring points have nitrate data from 9–26 years, the mean is 16 years, and 27% have data from more than 22 years.

**Figure 7:**
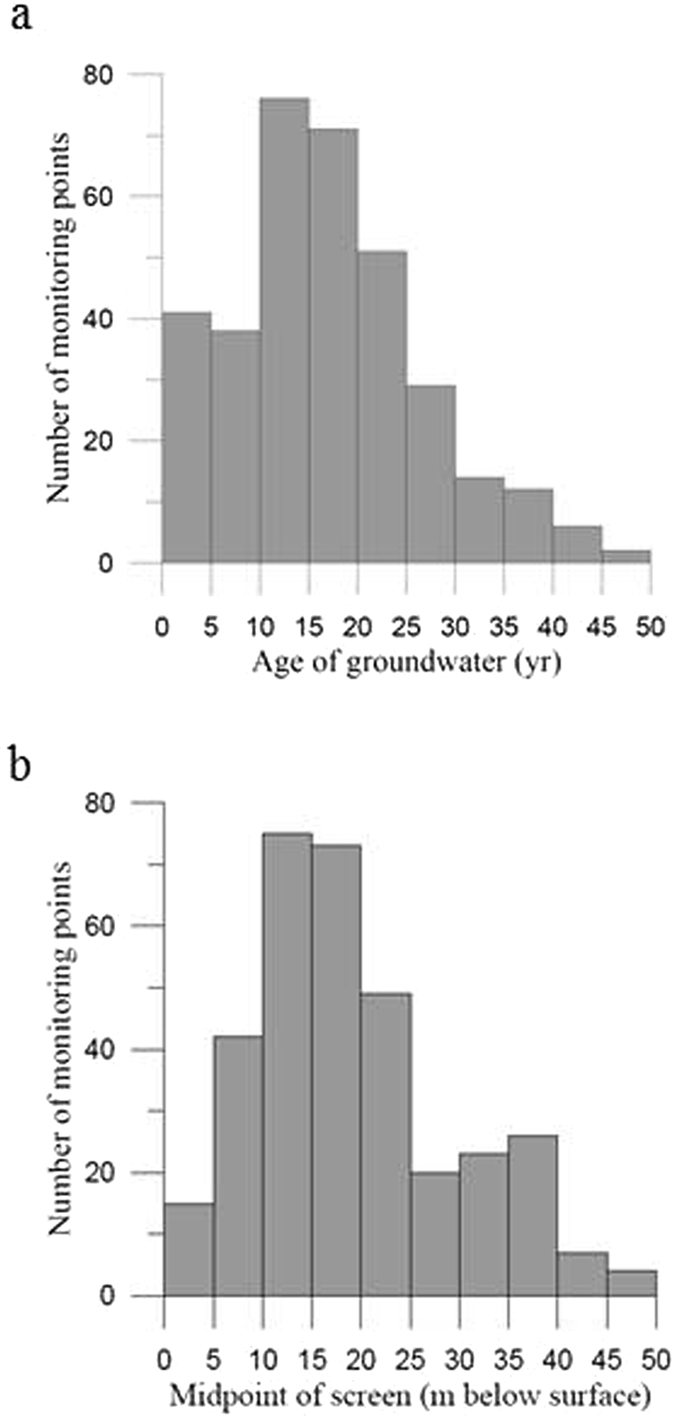
Distribution of the studied 340 monitoring points in Danish oxic groundwater according to the age of the groundwater (**a**) and the depth of the midpoint of the screen in m below surface (**b**).

Oxic groundwater in the 340 monitoring points has been dated at least once using either the CFC method (312 points during 1997–2013) or the tritium/helium method (28 points in 2013). The age of the groundwater in the monitoring points varies from 0.3–46 years (Fig. 5), and the mean and the median is approximately 17 years (Fig. 7a). The distribution of monitoring points (screens are usually short, approximately 1 m) with depth varies from a few meters to up to 50 meter below surface with a mean and median value of approximately 20 and 18 m below surface, respectively (Fig. 7b).

### Agricultural N balances

The difference between agricultural national N inputs (synthetic fertilizer, import of animal feed, organic waste products, net atmospheric deposition and fixation) and N outputs (plant and animal products) is defined as the N-surplus in agriculture at a national level^32^. The surplus is an overall environmental indicator of the changes in the agricultural impact on the environment and represents the amount of N not being used by the production system and therefore at risk of being lost to the environment.

The relation between the accounted agricultural national N outputs and N inputs is defined as the N use efficiency (NUE) of the whole agricultural system including both plant and animal production

Values for annual national N inputs and outputs in the primary Danish agricultural sector from 1941–2012 are based on data from Statistics Denmark and literature values^32, 52, 53^.

### Socio-economic drivers of environmental quality

Nitrate pollution levels of the groundwater were evaluated against an economic growth indicator, national Gross Domestic Product (GDP), to test the evidence of an environmental Kuznets curve (EKC), as recently demonstrated at the global level for N surplus by Zhang *et al*.^2^.

Data on the Danish annual GDP from 1966–2012 (current prices) was downloaded from Statistics Denmark. GDP values from 1948–1967 are available in a report from Statistics Denmark^54^.

### Statistical methods

Trend analysis of nitrate time series in groundwater wells at each monitoring point was performed as a simple linear regression with PROC REG from the SAS software system^55^. Distribution of trends between monitoring points grouped according to the groundwater infiltration time were compared in a regression model with separate regression lines for each of four infiltration periods and fitted as a random coefficient model with SAS PROC MIXED to allow for repeated measurements at each monitoring point.

For each model, probability plots of the residuals were checked for normality. Concerning the time series analysis in each of the 250 monitoring points in oxic groundwater, the series of residuals of the concentrations at each monitoring point were considered typical for normally distributed data based on scatter and normal probability plots. Overall the time series analyses at each monitoring points were found to meet the assumptions for linear regression.
